# Characterization of cardiac bradyarrhythmia associated with LGI1-IgG autoimmune encephalitis

**DOI:** 10.3389/fimmu.2022.948479

**Published:** 2022-10-11

**Authors:** Hannah H. Zhao-Fleming, Anza Zahid, Tong Lu, Xiaojing Sun, Sean J. Pittock, Hon-Chi Lee, Divyanshu Dubey

**Affiliations:** ^1^ Department of Neurology, Mayo Clinic, Rochester, MN, United States; ^2^ Department of Cardiovascular Medicine, Mayo Clinic, Rochester, MN, United States; ^3^ Department of Laboratory Medicine and Pathology, Mayo Clinic, Rochester, MN, United States

**Keywords:** LGI1-IgG, autoimmune encephalitis, cardiac bradyarrhythmia, outcomes, seizures

## Abstract

**Objective:**

To evaluate and characterize cardiac arrythmias associated with LGI1-IgG (Leucine-rich glioma inactivated 1–IgG) autoimmune encephalitis (AE).

**Patients and methods:**

In this retrospective descriptive study, we identified Mayo Clinic patients (May 1, 2008 – December 31, 2020) with LGI1-IgG AE who had electrocardiogram proven bradyarrhythmias during the initial presentation. Inclusion criteria were 1) LGI1-IgG positivity with a consistent clinical syndrome; 2) electrocardiographic evidence of bradyarrhythmia; and 3) sufficient clinical details. We excluded patients who were taking negative ionotropic agents at the time of their bradyarrhythmias. We collected demographic/clinical data including details of bradyarrhythmia (severity, duration, treatments), and neurologic and cardiac outcomes.

**Results:**

We found that patients with LGI1-IgG AE had bradyarrhythmia at a frequency of 8% during the initial presentation. The bradyarrhythmia was often asymptomatic (6/11, 55%); however, the episode was severe with one patient requiring a pacemaker. Outcome was also generally favorable with the majority (8/11, 73%) having full resolution without further cardiac intervention. Lastly, we found that mouse and human cardiac tissues express LGI1 (mRNA and protein).

**Conclusion:**

LGI1-IgG AE can be rarely associated with bradyarrhythmias. Although the disease course is mostly favorable, some cases may require pacemaker placement to avoid devastating outcomes.

## Introduction

A population-based epidemiology study based in Olmsted County demonstrated that autoimmune encephalitis is as common as infectious encephalitis. (1) Furthermore, over the last few decades recognition of this potentially treatable conditions has exponentially increased, primarily because of widespread utilization of neural autoantibody evaluation as diagnostic markers. Leucine-rich glioma inactivated 1 (LGI1) IgG is one of the most common pathogenic neural specific autoantibodies associated with autoimmune encephalitis in adults. (2) Few cases of bradyarrhythmia in association with LGI1 autoimmune encephalitis have been reported. (3, 4) However, larger cohort studies analyzing cardiac rhythm dysfunction are lacking, suggesting that this may be an under reported phenomenon. In this study, we characterize the episodes of bradyarrhythmias among LGI1 encephalitis patients and evaluate LGI1 expression in cardiac tissue.

## Methods

### Study approval and patient consents

The Mayo Clinic Institutional Review Board approved this study and all patients consented to the use of their medical records for research purposes.

### Patient identification

**Figure 1:**
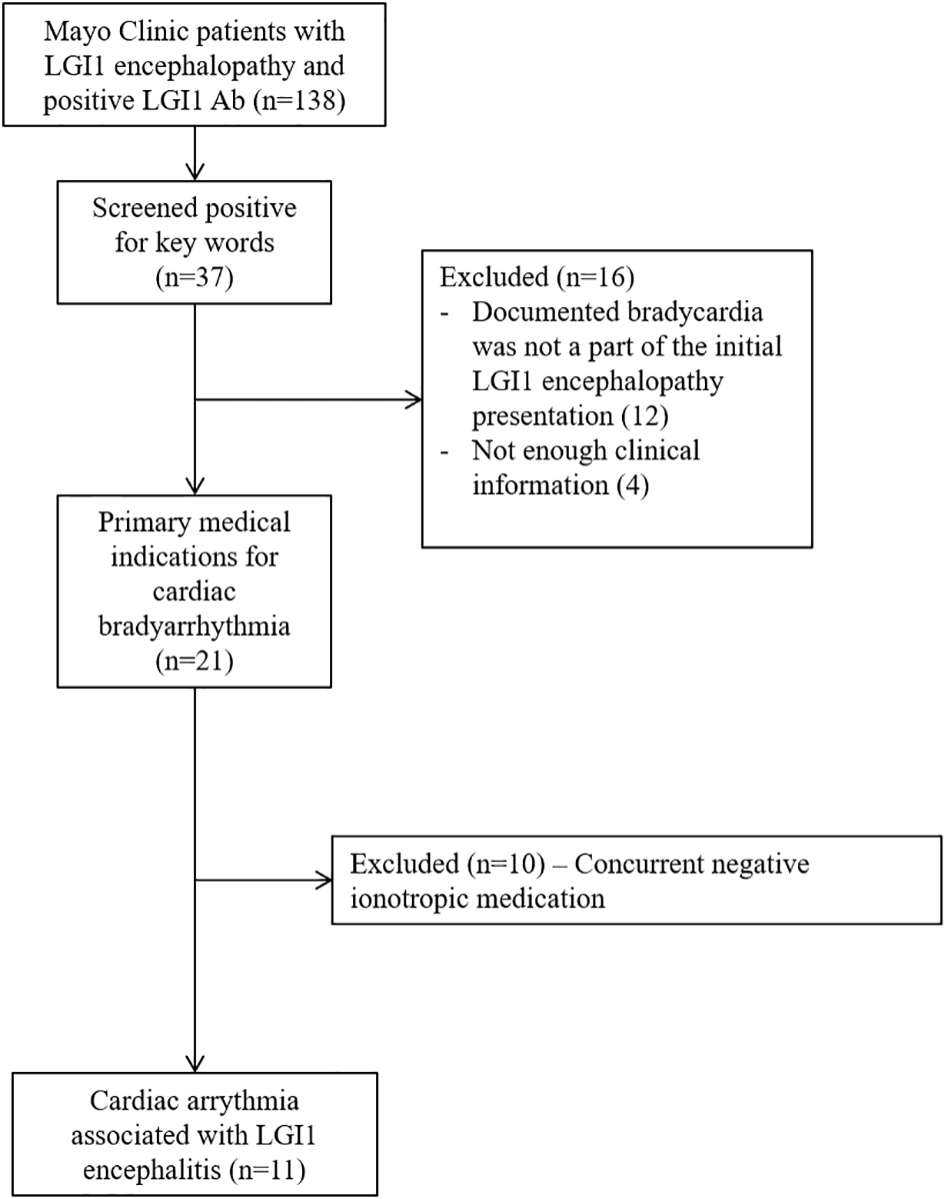
Flowchart depicting search strategy for identification of patients cardiac arrythmia within 6 months of LGI1 neurological autoimmunity.

We retrospectively identified Mayo Clinic patients from May 1, 2008 to December 31, 2020 through Advanced Cohort Explorer, an electronic retrieval system that interrogates the electronic medical record, and this was cross referenced with our prior studies on LGI1 antibody encephalitis. The inclusion criteria included: (1) LGI1 antibody positivity in serum and/or CSF; (2) patient evaluated at Mayo Clinic (Minnesota, Florida, Arizona); (3) clinical syndrome consistent with LGI1 IgG autoimmunity; and (4) electrocardiographic (ECG) documentation of bradycardia (defined as heart rate less than 60 beats per minute). Our exclusion criteria was 1) when bradycardia was not a part of the LGI1 antibody encephalitis presentation and 2) cases where there was concurrent negative ionotropic agents used. These medications include diltiazem, amiodaron, propranolol, metoprolol, midazolam and carvedilol. Mayo Clinic medical records of all included cases were reviewed by three physicians (H.H.Z., A.Z., and D.D.) independently. Detailed cohort identification process is outlined in **Figure 1**. Demographic and clinical data along with MRI brain, electrocardiography findings and clinical outcomes were collected.

### LGI1-IgG assay

LGI1 autoantibodies were detected by transfected cell-based immunofluorescence assay (CBA; EUROIMMUN, Lubeck, Germany) in Mayo Neuroimmunology Laboratory, as previously described (5).

### Quantitative real-time PCR

mRNA was extracted from isolated C57BL/6 (The Jackson Laboratory) mouse cardiomyocytes with the RNeasy Plus Mini Kit (Qiagen, Cat. No: 74134). The mRNA was then reverse transcribed to cDNA using SuperScript™ III First-Strand Synthesis SuperMix (Invitrogen, Cat. No: 18080-400). This cDNA is then amplified using taq polymerase (Denville Scientific, Cat. No: 1091201), and PCR primer: LGI-1 primers: 5’ TCC TCG AAG GAT TTC GAT TG 3’(forward), 5’ ACA TGG TCC CAT TCA AGG AA 3’(reverse). GAPDH primers: 5′-TGCCAAGGCTGTGGGCAAGG-3′ (forward) and 5′-TGGGCCCTCAGATGCCTGCT-3′ (reverse). Thermocycling was done as follows: initial denaturation at 94°C for 5 minutes, 35 cycles of denaturation at 94°C for 30 seconds, primer annealing at 58°C for 30 seconds, and extension at 72°C for 30 seconds, followed by final extension at 72°C for 5 minutes.

### Immunoprecipitation

Human heart tissue from an organ donor was ground into powder with mortar and pestle in liquid nitrogen. This was made into a solution with RIPA buffer (in mM): Tris 50, NaCl 150, NaF 2, EDTA 1, EGTA 1, NaVO4 1, and 1% Triton X-100 containing protease inhibitor cocktail (Complete Mini, EDTAfree; Roche Diagnostics GmbH, Germany) and placed on ice for 1 hour. Then, the homogenate was centrifuged at 8700 rpm at 4°C for 10 min. The supernatant (200 μg in 200 μl) was pre-cleared with Protein G Plus-agarose beads (Santa Cruz Biotechnology, Santa Cruz, CA) at 4°C for 1 hour, and the pre-cleared supernatant was incubated with mouse anti-LGI1 antibodies (2 µg/200 µg protein, Invitrogen, S283-7) at 4°C overnight. The next day, the samples were incubated with 30 μl Protein G Plus-agarose at 4°C for 3 h with rotation. After washing the beads with RIPA/protease inhibitor buffer, the immunoprecipitates were collected and eluted with 30 μl SDS-PAGE loading buffer per tube. The immunoprecipitates were resolved by SDS-PAGE (4–15% gel) and blotted against anti-LGI1 antibody with 1:1000 dilution.

## Results

### Patient cohort and frequency of cardiac arrythmia

Among 137 cases we found 11 cases with LGI1 IgG seropositivity to have a cardiac bradyarrhythmia that is not otherwise explained by medical co-morbidities, representing a frequency of 8%. The median age of our cohort was 64 years old (range 18-82 years), and 8/11 (73%) of our cohort was male. In our cohort, (121/137, 88%) had cardiac rhythm measured by ECG, (71/121, 59%) with 12-lead ECG, and (50/121, 41%) with 1-2 lead ECG within an electroencephalogram (EEG) evaluation. Of the 50 patients whose cardiac rhythm was monitored only within the EEG, only 8 (16%) had EEG reports that commented on the heart rate specifically. Only two of these 11 patients had history of structural heart disease (one had coronary artery disease and the other had mitral regurgitation). Other medical comorbidities included hypertension (n=7), hyperlipidemia (n=6), diabetes mellitus (n=3) and obstructive sleep apnea (n=2). Four patients had inhouse echocardiograms done after the bradycardia event, none of which demonstrated any ischemic cardiac changes. Two patients had neurology notes mention outside echocardiogram performed and was unremarkable. Five patients did not have internal or external echocardiograms. Three of our patients had a history of autoimmunity (Hashimoto thyroiditis, prior acute inflammatory demyelinating polyneuropathy, and interstitial lung disease). Three of our patients had a history of cancers, none of which were active at the time of LGI1 encephalitis (prostate cancer and melanoma, prostate cancer and basal cell carcinoma of the skin, and squamous cell carcinoma of the skin). Further details of our patient population, as well as certain details of their cardiac episode are described in **Table 1**.

**Table 1:** Demographics, co-morbidities, details of cardiac episode, treatments and outcomes of symptomatic bradycardiac associated with LGI1 encephalopathy.

LGI1-IgG positive patients with bradycardia	(n = 11)
** Demographics**	
Age at onset – Median (range, years)	64 (18-82)
- Sex, male (%)	8/11 (73%)
- Ethnicity, Caucasian (%)	11/11 (100%)
**Sodium levels**	
- Hyponatremia^a^ anytime during episode	7/11 (64%)
- Lowest sodium level during episode – Median (range, mmol/L)	131 (114-138)
- Hyponatremia at time of bradycardia capture^b^	1/10 (10%)
**ECG findings**	
- Sinus bradycardia (%)	7/11 (64%)
- Sinus bradycardia with PVCs (%)	2/11 (18%)
- Bradycardia with 1st degree AV block (%)	1/11 (9%)
**EEG findings^c^**	
- Ictal discharges (%)	5/10 (50%)
- Interictal discharges/slowing (%)	2/10 (20%)
- Normal (%)	3/10 (30%)
**Bradycardia onset/detection**	
- After onset of seizures	5/11 (45%)
- Preceding onset of seizures	3/11 (27%)
- During same presentation	3/11 (27%)
**MRI findings**	
- Medial temporal T2 hyperintensity, n (%)	5/11 (36%)
- Other abnormalities	2/11 (18%)
- Unremarkable findings (%)	4/11 (36%)
**Immunotherapies**	
- Corticosteroids	6/11 (55%)
- PLEX	0/11 (0%)
- IVIG	2/11 (18%)
**Cardiac Outcome**	
- No evidence of continued bradycardia	8/11 (73%)
- Continued bradycardia	1/11 (9%)
- Pacemaker placement	1/11 (9%)
**Neurologic outcome at last follow-up**	
- Seizure free on antiepileptic	3/11 (27%)
- Seizure free on immunomodulation	4/11 (36%)
- Residual cognitive deficits	3/11 (27%)
- Persistent seizures and cognitive decline	1/11 (9%)
- Unknown	2/11 (18%)

^a^Hyponatremia defined as serum sodium levels less than 135 mmol/L, ^b^One patient did not have sodium documented at bradycardia capture, ^c^One patient without EEG documented.

AV, atrial ventricular; ECG, electrocardiogram; EEG, electroencephalogram; IVIG, intravenous immunoglobulins; PLEX, plasma exchange; PVC, premature ventricular contraction.

### Clinical information of the cardiac arrythmia event

In all 11 patients, cardiac arrhythmia was a part of their initial presentation. Although hyponatremia was common during the encephalitic episode (64%), it was not common at the time of bradycardia capture (10%, **Table 1**). When available, other electrolytes were also within normal levels (potassium levels available in 10/11 patients and magnesium levels available in 3/11 patients). Neurological presentation was most commonly seizures (6/11, 55%), followed by encephalopathy (2/11, 18%) and both seizures and encephalopathy (2/11, 18%), and one patient presented with neuropathic pain involving the lower extremities. All the cardiac episodes were associated with seizures within 8 months. Seizure semiology was most commonly focal sensory aware (4/11, 36%), followed by focal sensory with impaired awareness (2/11, 18%), focal motor aware (2/11, 18%), focal motor/sensory with impaired awareness (2/11, 18%), and focal motor/sensory aware (1/11, 9%). In 6/11 (55%) of our patients, faciobrachial dystonic seizures were additionally noted. EEG showed interictal discharges or slowing in 2/10 patients (one patient had bi-hemispheric slowing and the other had epileptiform changes in the right temporal head region) and temporal lobe ictal discharges in 5/10 patients. Three patients had other documented evidence of autonomic dysfunction (all orthostatic dysfunction), with one patient having objective evidence of orthostatic intolerance on autonomic reflex testing. Notably, at the time of the bradycardia capture, 6 patients were taking antiseizure medications, 5 of whom were taking sodium channel blockers, specifically lacosamide, valproic acid, phenytoin, and oxcarbazepine. The one patient in our cohort who had persistent bradycardia was on phenytoin during initial bradycardia episode.

ECG findings of bradyarrhythmia were most commonly asymptomatic (6/11, 55%), median heart rate on initial ECG was 55 (range 37-59). In our patients who were symptomatic in association with their bradyarrhythmia, symptoms were either mild (generalized tremulousness) or more severe (presyncope, syncope, and exercise intolerance). In one of our patients, the bradycardia led to sinus arrest, requiring placement of a pacemaker, 2 months prior to the development of encephalopathy and subsequent LGI1-IgG evaluation. One patient was noted to have bradycardia during routine EEG without associated ictal discharges.

Most patients had favorable outcomes from neurological perspective. All patients achieved seizure freedom (**Table 1**). Three of our patients had continued cognitive deficits, including memory deficits and word finding difficulties. Two patients were still on a corticosteroid taper at last follow-up with cognitive deficits thought to be secondary to the active autoimmune encephalitis, while one patient had persistent cognitive deficit, thought to be the sequelae of prior encephalitis and immunomodulation was not offered.

From a cardiac perspective, one patient required pacemaker placement and another patient had persistent asymptomatic bradycardia at last follow-up 9 months after initial presentation, but she did not require additional management. Lastly, another LGI1 encephalitis patient who had arrythmia at the time of initial presentation, presented to the emergency department at an outside hospital with syncopal episodes, followed by unexpected sudden death three days later.

### LGI1 expression *in vivo*

**Figure 2:**
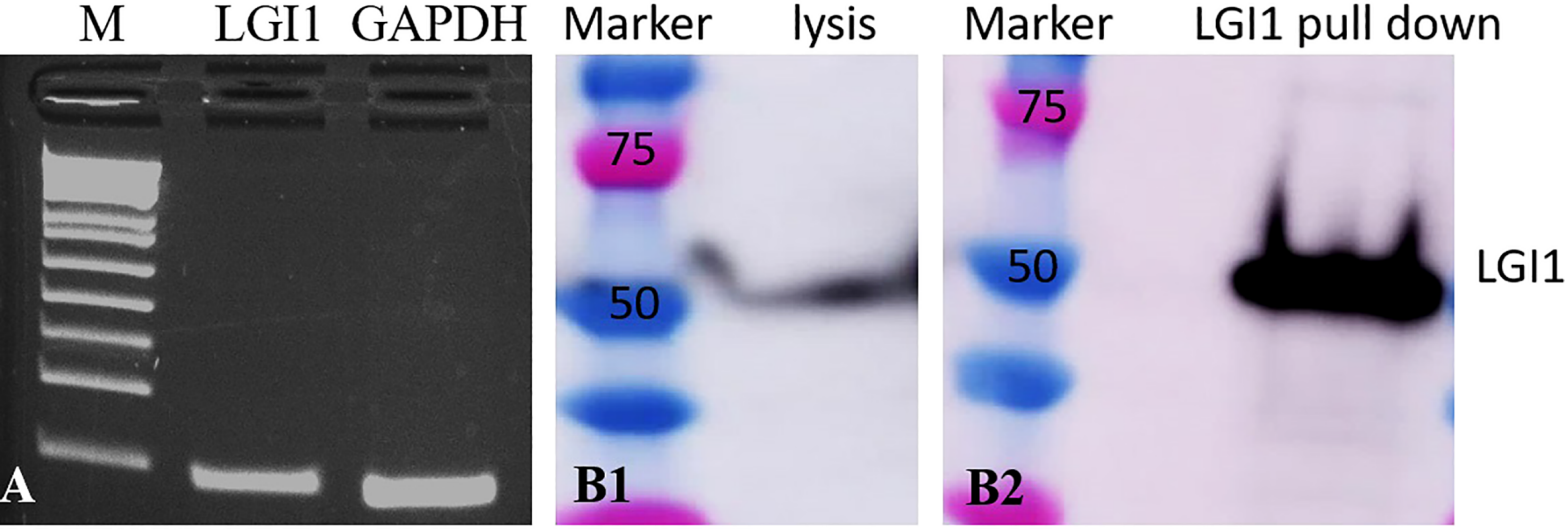
Cardiomyocytes express LGI1 mRNA and protein. **(A)** qt-PCR showing LGI1 and GAPDH (control) mRNA expression in mouse cardiomyocytes; **(B)** western blot of human heart ventricle lysate demonstrates binding of commercial LGI1 commercial antibodies to ~50 kD protein (B1); immunoprecipitation and western blot screening of human heart ventricle lysates with LGI1 commercial antibody also demonstrates a ~50 kD molecular weight band (B2).

We found that LGI1 mRNA is expressed in wild type mouse cardiomyocytes by RT-PCR (**Figure 2A**). We also found that LGI1 protein is expressed in human cardiomyocytes *via* immunoprecipitation and western blotting (**Figure 2B**).

## Discussion

In this study, we described a cohort of LGI1-IgG seropositive patients who had bradycardia at presentation. Few cases had bradycardia preceding the onset of encephalopathy. The outcomes of the bradycardia were generally positive, with 73% recovering completely. One patient had continued bradycardia without the need for further intervention, and another patient required pacemaker placement. The general demographics, including age, sex, and ethnicity of the cases included in this study are like prior studies, (6) suggesting that this is not a sub-specific group pre-disposed to cardiac arrythmias. The incidence of sinus bradycardia in our cohort is 8% and appears to be higher than that of the general population which is estimated to be 0.8 per 1000 person-year, although the true incidence of sinus node dysfunction in the general population is unknown. (7, 8)

Several mechanisms for cardiac arrythmias associated with seizure disorders have been proposed in the past, such as temporal lobe seizures causing ictal bradycardia (9) and involvement of the insular cortex contributing to bradyarrhythmias (10). Even among the cases included in this study we cannot exclude effect of temporal or insular discharges on cardiac arrythmias. However, as all cardiac dysrhythmias in our study were not temporally related to the seizures an alternative pathophysiology such as a potential direct role of LGI1 IgG on cardiac myocytes is possible. LGI1, an extracellular component of the voltage-gated potassium channel (VGKC) complex, is tightly complexed with Kv1 channels and LGI1 antibodies are thought to bind to LGI1 and alter neuronal excitability and synaptic transmission. (11) LGI1 has been reported to co-assemble with Kv1.4 and KVβ1, slowing channel inactivation through inhibition of the effects of Kvβ1. (12) It is intriguing that Kv1.4 is known to be present in the heart. (13) Whether LGI1 antibodies would affect the function of Kv1.4 in the heart is unknown. Our finding that LGI1 is expressed in cardiac tissue raises the possibility of direct cardiac effect of the LGI1-IgG as a potential etiology for these cardiac manifestations.

Patients with asymptomatic sinus bradycardia in the general population are not associated with incident cardiovascular diseases or mortality. (14) Although most of our patients had favorable cardiac outcomes, one patient with focal motor seizures presented to the emergency department with a syncopal episode while corticosteroids were being tapered. Due to lack of access to outside facility records presence of persistent cardiac arrythmia could not be confirmed at/around the time of syncope. He unfortunately died suddenly three days later without a clear cause.

Sudden cardiac death has also been previously reported in LGI1 encephalitis. (15) In the reported case, no arrythmia was documented because the event happened outside the hospital setting. Pathology showed no significant coronary disease or evidence of myocardial ischemia, further highlighting the importance of understanding the rare but potential fatal cardiac dysfunction in LGI1 AE. Furthermore, in a large study on sudden death in epilepsy, the authors found that heart rate variability derangement is significantly more pronounced in epilepsy patients prior to their sudden death when compared with the surviving epileptic controls, suggesting a strong cardiac rhythm component in sudden death in epilepsy. (16)

Studies have demonstrated that the anti-seizure medications (ASMs) working primarily through sodium channel blockade may be more efficacious than other ASMs with other mechanisms of action for managing seizures associated with LGI1 AE. (17) However, some sodium channel blocking medications, such as lacosamide, can cause or potentially worsen bradyarrhythmias. (18, 19) Sodium channel blocking drugs are known to be proarrhythmic in patients with history of coronary artery disease and structural heart disease. (20) Indeed, five of our patients were taking sodium channel blockers during their bradycardia episode, therefore among these cases adverse effect of anti-seizure medications cannot be completely excluded. Pre-screening LGI1 AE patients with an electrocardiogram may help identify patients with bradyarrhythmias, which will aid in the ASM selection.

Our study has some potential limitations including its retrospective design. There is likely under detection of cardiac arrythmias if the patient did not exhibit overt cardiac symptoms such as syncope prompting an ECG. Indeed, only 71/137 (52%) of our cohort had their cardiac rhythms accessed by a 12-lead ECG at the time of their initial presentation. Although more of our cohort did have heart rate monitoring as a part of their EEG study, only 16% of the EEG reports commented on heart rates in particular. Lastly, our cohort was 73% male with a median age of 64 years, which is the demographic prone to cardiovascular comorbidities. (21) Although we excluded patients with known alternate cardiac causes for arrhythmias, we cannot exclude the possibility that our patients had preclinical or undiagnosed cardiac comorbidities. Indeed, five of our patients did not have any cardiac echocardiogram documented after their bradycardiac episode. However, temporal association of detection of arrythmias with onset of neurological dysfunction and demonstration of LGI1 protein expression in the cardiac tissue supports the presence of this rare but potentially fatal cardiac manifestation of LGI1 autoimmunity.

## Data availability statement

The raw data supporting the conclusions of this article will be made available by the authors, without undue reservation.

## Ethics statement

The studies involving human participants were reviewed and approved by Mayo Clinic Institutional Review Board. The patients/participants provided their written informed consent to participate in this study.

## Author contributions

HHZ designed and conceptualized study; drafted the manuscript and figures; analyzed and interpreted the data. AZ analyzed and interpreted the data; revised the manuscript for intellectual content. TL performed RT-PCR and immunoprecipitation experiments; Interpreted the data; revised the manuscript for intellectual content. XS performed RT-PCR and immunoprecipitation experiments; Interpreted the data; revised the manuscript for intellectual content. SP interpreted the data; revised the manuscript for intellectual content. H-CL designed and conceptualized study; interpreted the data; revised the manuscript for intellectual content. DD designed and conceptualized study; analyzed and interpreted the data; Revised the manuscript for intellectual content; study supervision. All authors contributed to the article and approved the submitted version.

## Conflict of interest

SP reports grants, personal fees and non-financial support from Alexion Pharmaceuticals, Inc.; grants, personal fees, non-financial support and other support from MedImmune, Inc/Viela Bio.; personal fees for consulting from Genentech/Roche. He has a patent, Patent# 8,889,102 (Application#12-678350, Neuromyelitis Optica Autoantibodies as a Marker for Neoplasia) – issued; a patent, Patent# 9,891,219B2 (Application#12-573942, Methods for Treating Neuromyelitis Optica [NMO] by Administration of Eculizumab to an individual that is Aquaporin-4 (AQP4)-IgG Autoantibody positive) – issued. DD has consulted for UCB, Immunovant and Astellas pharmaceuticals. All compensation for consulting activities is paid directly to Mayo Clinic. DD has patents pending for KLHL11 and LUZP4 as markers of neurological autoimmunity.

The remaining authors declare that the research was conducted in the absence of any commercial or financial relationships that could be construed as a potential conflict of interest

## Publisher’s note

All claims expressed in this article are solely those of the authors and do not necessarily represent those of their affiliated organizations, or those of the publisher, the editors and the reviewers. Any product that may be evaluated in this article, or claim that may be made by its manufacturer, is not guaranteed or endorsed by the publisher.

## References

[1] Dubey D Pittock SJ Kelly CR Mckeon A Lopez-Chiriboga AS Lennon VA. Autoimmune encephalitis epidemiology and a comparison to infectious encephalitis. Ann Neurol (2018) 83:166–77. doi: 10.1002/ana.25131 PMC6011827 29293273

[2] Binks SNM Klein CJ Waters P Pittock SJ Irani SR. LGI1, CASPR2 and related antibodies: a molecular evolution of the phenotypes. J Neurol Neurosurg Psychiatry (2018) 89:526–34. doi: 10.1136/jnnp-2017-315720 PMC5909759 29055902

[3] Naasan G Irani SR Bettcher BM Geschwind MD Gelfand JM. Episodic bradycardia as neurocardiac prodrome to voltage-gated potassium channel complex/leucine-rich, glioma inactivated 1 antibody encephalitis. JAMA Neurol (2014) 71:1300–4. doi: 10.1001/jamaneurol.2014.1234 PMC4474144 25133690

[4] Nilsson AC Blaabjerg M. More evidence of a neurocardiac prodrome in anti-LGI1 encephalitis. J Neurol Sci (2015) 357:310–1. doi: 10.1016/j.jns.2015.07.015 26194046

[5] Klein CJ Lennon VA Aston PA Mckeon A O’toole O Quek A. Insights from LGI1 and CASPR2 potassium channel complex autoantibody subtyping. JAMA Neurol (2013) 70:229–34. doi: 10.1001/jamaneurol.2013.592 PMC3895328 23407760

[6] Dubey D Britton J Mckeon A Gadoth A Zekeridou A Lopez Chiriboga SA. Randomized placebo-controlled trial of intravenous immunoglobulin in autoimmune LGI1/CASPR2 epilepsy. Ann Neurol (2020) 87:313–23. doi: 10.1002/ana.25655 PMC7003900 31782181

[7] Tsao CW Aday AW Almarzooq ZI Alonso A Beaton AZ Bittencourt MS. Heart disease and stroke statistics-2022 update: A report from the American heart association. Circulation (2022) 145:e153–639. doi: 10.1161/CIR.0000000000001052 35078371

[8] Jensen PN Gronroos NN Chen LY Folsom AR Defilippi C heckbert SR. Incidence of and risk factors for sick sinus syndrome in the general population. J Am Coll Cardiol (2014) 64:531–8. doi: 10.1016/j.jacc.2014.03.056 PMC4139053 25104519

[9] Britton JW Ghearing GR Benarroch EE Cascino GD. The ictal bradycardia syndrome: localization and lateralization. Epilepsia (2006) 47:737–44. doi: 10.1111/j.1528-1167.2006.00509.x 16650140

[10] Oppenheimer SM Gelb A Girvin JP Hachinski VC. Cardiovascular effects of human insular cortex stimulation. Neurology (1992) 42:1727–32. doi: 10.1212/WNL.42.9.1727 1513461

[11] van Sonderen A Petit-Pedrol M Dalmau J Titulaer MJ. The value of LGI1, Caspr2 and voltage-gated potassium channel antibodies in encephalitis. Nat Rev Neurol (2017) 13:290–301. doi: 10.1038/nrneurol.2017.43 28418022

[12] Schulte U Thumfart JO Klöcker N Sailer CA Bildl W Biniossek M. The epilepsy-linked Lgi1 protein assembles into presynaptic Kv1 channels and inhibits inactivation by Kvbeta1. Neuron (2006) 49:697–706. doi: 10.1016/j.neuron.2006.01.033 16504945

[13] Grandi E Sanguinetti MC Bartos DC Bers DM Chen-Izu Y Chiamvimonvat N. Potassium channels in the heart: structure, function and regulation. J Physiol (2017) 595:2209–28. doi: 10.1113/JP272864 PMC5374109 27861921

[14] Dharod A Soliman EZ Dawood F Chen H Shea S Nazarian S. Association of asymptomatic bradycardia with incident cardiovascular disease and mortality: The multi-ethnic study of atherosclerosis (MESA). JAMA Intern Med (2016) 176:219–27. doi: 10.1001/jamainternmed.2015.7655 26785103

[15] Rizzi R Fisicaro F Zangrandi A Ghidoni E Baiardi S Ragazzi M. Sudden cardiac death in a patient with LGI1 antibody-associated encephalitis. Seizure (2019) 65:148–50. doi: 10.1016/j.seizure.2019.01.013 30708290

[16] Sivathamboo S Friedman D Laze J Nightscales R Chen Z Kuhlmann L. Association of short-term heart rate variability and sudden unexpected death in epilepsy. Neurology (2021) 97:e2357–67. doi: 10.1212/WNL.0000000000012946 34649884

[17] Feyissa AM López-Chiriboga AS Britton J. W. Antiepileptic drug therapy in patients with autoimmune epilepsy. Neurol Neuroimmunol Neuroinflamm (2017) 4:e353. doi: 10.1212/NXI.0000000000000353 28680914 PMC5489139

[18] Nizam A Mylavarapu K Thomas D Briskin K Wu B Saluja D. Lacosamide-induced second-degree atrioventricular block in a patient with partial epilepsy. Epilepsia (2011) 52:e153–5. doi: 10.1111/j.1528-1167.2011.03212.x 21801173

[19] Yang C Peng Y Zhang L Zhao L. Safety and tolerability of lacosamide in patients with epilepsy: A systematic review and meta-analysis. Front Pharmacol (2021) 12:694381. doi: 10.3389/fphar.2021.694381 34616294 PMC8488108

[20] Roden DM. Pharmacology and toxicology of Nav1.5-class 1 anti-arrhythmic drugs. Card Electrophysiol Clin (2014) 6:695–704. doi: 10.1016/j.ccep.2014.07.003 25395995 PMC4226533

[21] Khurshid S Choi SH Weng L-C Wang EY Trinquart L Benjamin EJ. Frequency of cardiac rhythm abnormalities in a half million adults. Circulation: Arrhythmia Electrophysiology (2018) 11:e006273. doi: 10.1161/CIRCEP.118.006273 29954742 PMC6051725

